# Differences between men and women accessing an Australian perinatal and infant mental health care navigation service—Why do fathers seek help?

**DOI:** 10.1002/imhj.70012

**Published:** 2025-03-19

**Authors:** Sophia A. Harris, Valsamma Eapen, Jane Kohlhoff

**Affiliations:** ^1^ Discipline of Psychiatry & Mental Health University of New South Wales Sydney Australia; ^2^ Academic Unit of Infant, Child & Adolescent Psychiatry Services (AUCS) South Western Sydney Local Health District and Ingham Institute Sydney Australia; ^3^ Research Department Karitane Sydney Australia

**Keywords:** fathers, paternal, perinatal mental health, الآباء، التربية الوالدية، الصحة النفسية في الفترة المحيطة بالولادة, 父亲群体, 父亲的, 围产期心理健康, Père, paternel, santé mentale périnatale, Väter, väterlich, perinatale psychische Gesundheit, 父親、父性、周産期メンタルヘルス, papás, paterno, salud mental perinatal

## Abstract

This study explored the demographic and psychosocial characteristics, and presenting concerns of new or expectant fathers seeking perinatal mental health (PMH) support through the Australia‐based ForWhen service, compared to a sample of mothers. The retrospective observational analysis examined routinely collected data from 105 male and 203 female clients who were supported by ForWhen between February 2022 to June 2024. Fathers and mothers did not differ in terms of demographic characteristics, and both presented with similarly high levels of distress during intake. However, fathers were more likely to report current self‐harm and/or suicidal ideation, as well as current relationship issues and financial stress. Conversely, mothers were more likely to report parenting concerns such as infant sleep and settling challenges. Overall, far fewer men than women access support through ForWhen, despite the known prevalence of PMH concerns among fathers. There were also differences in how clients accessed the service, with fathers more often referred by their intimate partner, suggesting that partners may be an important avenue to encourage help‐seeking for paternal PMH concerns. These findings highlight the need to adapt PMH services—traditionally designed for women—to be more inclusive of and better engage men.

## ملخص

تناولت هذه الدراسة الخصائص الديموغرافية والنفسية الاجتماعية والشواغل الحالية للآباء الجدد الذين يسعون للحصول على دعم الصحة النفسية في الفترة المحيطة بالولادة من خلال خدمةForWhen في أستراليا، مقارنة بعينة من الأمهات. تم مراجعة البيانات بأثر رجعي والتي تم جمعها بشكل روتيني من العملاء (105 من الرجال و203 من السيدات) الذين تلقوا الدعم من خلال خدمةForWhen في الفترة ما بين فبراير 2022 إلى يونيو 2024. لم يختلف الآباء والأمهات من حيث الخصائص الديموغرافية، وكلهم أظهروا لمستويات عالية مماثلة من الضيق أثناء الالتحاق بالعيادة. ومع ذلك، كان الآباء أكثر عرضة للإبلاغ عن إيذاء النفس الحالي و/أو التفكير في الانتحار، بالإضافة إلى مشاكل العلاقة الحالية والضغوط المالية. وعلى العكس من ذلك، كانت الأمهات أكثر عرضة للإبلاغ عن مخاوف التربية مثل مشاكل نوم الرضع وتحديات الاستقرار. بشكل عام، كان عدد الرجال الذين يحصلون على الدعم من خلال برنامجForWhen أقل بكثير من النساء، على الرغم من الانتشار المعروف لمخاوف الصحة النفسية الأبوية بين الآباء. كانت هناك أيضًا اختلافات في كيفية وصول العملاء إلى الخدمة، حيث تمت إحالة الآباء في كثير من الأحيان من قبل الشريك، مما يشير إلى أن الشركاء قد يكونون وسيلة مهمة لتشجيع طلب المساعدة في مخاوف الصحة النفسية الأبوية في الفترة المحيطة بالولادة. تسلط هذه النتائج الضوء على الحاجة إلى تطويع خدمات الصحة النفسية في الفترة المحيطة بالولادة—المصممة تقليديًا للنساء—لتكون أكثر شمولاً للرجال وإشراكهم بشكل أفضل.

## 摘 要

本研究探讨了通过澳大利亚ForWhen服务寻求围产期心理健康支持的新手父亲或准父亲的人口统计学特征、社会心理特征以及主诉问题, 并与母亲样本进行对比。通过回顾性数据分析, 研究者审查了2022年2月至2024年6月期间接受ForWhen服务的105名男性客户与203名女性客户的常规收集数据。研究发现, 父亲和母亲在人口统计学特征上无显著差异, 且两者在初次评估时均表现出较高水平的心理困扰。然而, 父亲群体更有可能报告当前的自残或自杀意念、当前伴侣关系问题及经济压力。相比之下, 母亲群体更倾向于报告育儿方面的困扰, 如婴儿睡眠和安抚方面的困难。总体而言, 尽管已知父亲在围产期心理健康方面同样存在问题, 但通过ForWhen寻求支持的男性远少于女性。此外, 在获取服务的方式上也存在差异, 父亲更多通过其亲密伴侣转介, 这表明伴侣可能是鼓励男性寻求围产期心理健康支持的重要途径。这些发现强调需要改进传统以女性为中心的围产期心理健康服务体系, 使其更具包容性并有效吸引男性参与。

## Résumé

Cette étude a examiné les caractéristiques démographiques et psychosociales, et les préoccupations des nouveaux pères ou des futurs pères cherchant un soutien en santé mentale périnatale par l'intermédiaire du service australien ForWhen, avec une comparaison avec un échantillon de mères. L'examen rétrospectif des données a examiné les données recueillies de façon régulière auprès de 105 clients masculins et 203 clientes ayant été soutenus par ForWhen entre février 2022 et juin 2024. Les pères et les mères ne différaient pas en matière de caractéristiques démographiques, et tous présentaient des niveaux de détresse aussi élevésà l'admission. Cependant, les pères étaient plus à même de signaler des problèmes actuels d'automutilation et/ou d'idées suicidaires, ainsi que des problèmes relationnels et un stress financier. Inversement, les mères étaient plus à même de signaler des problèmes liés au parentage, comme le sommeil du nourrisson et les difficultés d'adaptation. Dans l'ensemble, beaucoup moins d'hommes que de femmes demande un soutien par le biais du ForWhen, malgré la prévalence connue des préoccupations liées au PMH chez les pères. On a aussi constaté des différences dans la façon dont les clients accédaient au service, les pères étant plus souvent référés par leur partenaire intime, ce qui suggère que le partenaire peut être un moyen important d'encourager la recherchede soutien pour les problèmes de santé mentale périnatale du père. Ces résultats soulignent la nécessité d'adapter les services de santé mentale périnatale—traditionnellement conçus pour les femmes—afin qu'ils soient plus inclusifs et impliquent mieux les hommes.

## Zusammenfassung

Diese Studie untersuchte die demografischen und psychosozialen Merkmale sowie die Vorstellungsgründe von neuen oder werdenden Vätern im Vergleich zu einer Stichprobe von Müttern, die beide im Rahmen des australischen ForWhen‐Dienstes Unterstützung im Bereich der perinatalen psychischen Gesundheit suchen. Die retrospektive Datenanalyse untersuchte routinemäßig erhobene Daten von 105 männlichen und 203 weiblichen Klient:innen, die zwischen Februar 2022 und Juni 2024 durch ForWhen unterstützt wurden. Väter und Mütter unterschieden sich nicht in Bezug auf demografische Merkmale und beide wiesen bei der Erstvorstellung ein ähnlich hohes Maß an Belastung auf. Allerdings berichteten Väter häufiger über aktuelles selbstverletzendes Verhalten und/oder Suizidgedanken sowie über aktuelle Beziehungsprobleme und finanziellen Stress. Umgekehrt berichteten Mütter häufiger von Erziehungssorgen wie Schlaf‐ und Eingewöhnungsproblemen bei Säuglingen. Insgesamt nehmen weitaus weniger Männer als Frauen Unterstützung durch ForWhen in Anspruch, obwohl bekannt ist, dass Väter häufiger von PMH betroffen sind. Es gab auch Unterschiede in der Art des Zugangs der Klient:innen: Väter wurden häufiger von Lebensgefährt:innen an ForWhen verwiesen, was darauf hindeutet, dass die Partner:innen ein wichtiger Weg sein könnten, um Hilfesuchenden bei perinatalen psychischen Problemen zu unterstützen. Diese Ergebnisse unterstreichen die Notwendigkeit, perinatale psychische Gesundheitsdienste—die traditionell für Frauen konzipiert sind—so anzupassen, dass sie Männer stärker einbeziehen und besser einbinden.

## 要旨

本研究では、オーストラリアを拠点とするForWhenサービスを通じて周産期メンタルヘルス支援を求める新米の父親あるいはこれから父親になる男性の、人口統計学的および心理社会的特徴、懸念事項を、母親のサンプルと比較して検討した。レトロスペクティブ・データ・レビューでは、2022年2月から2024年6月の間にForWhenの支援を受けた男性105人、女性203人の日常的に収集されたデータを調査した。父親と母親の人口統計学的特徴に差はなく、受け入れ時に両者とも同様に高いレベルの苦痛を示した。しかし、父親は、現在の自傷行為や自殺願望、また現在の人間関係の問題や金銭的なストレスを報告する傾向がより強かった。逆に、母親は乳児の睡眠やあやし方といった子育ての悩みを報告する傾向が強かった。父親の周産期メンタルヘルスに関する悩みの有病率が知られているにもかかわらず、ForWhenを通じて支援を受ける男性は、全体的に女性よりもはるかに少なかった。また、相談者のサービスへのアクセス方法にも違いがあり、父親の場合は親密なパートナーから紹介されることが多く、パートナーが父親の周産期のメンタルヘルス上の懸念に対して助けを求めることを促す重要な道筋となる可能性があることが示唆された。これらの調査結果は、従来女性向けに設計されていた周産期メンタルヘルスサービスを、男性にもより広く対応し、より積極的に関与してもらえるようにする必要性を強調している。

## Resumen

Este estudio exploró las características demográficas y sicosociales, así como presentar las inquietudes de nuevos papás y papás en espera de serlo que buscaron apoyo perinatal de salud mental por medio del servicio ParaCuando, con base en Australia, comparados con un grupo muestra de madres. La revisión de datos retrospectivos examinados recogió rutinariamente información de 105 clientes varones y 203 mujeres quienes recibieron apoyo de ParaCuando entre febrero de 2022 y junio de 2024. Los papás y las mamás no difirieron en términos de características demográficas, y ambos presentaron similarmente altos niveles de angustia durante el proceso de apoyo. Sin embargo, los papás estuvieron más propensos a reportar presentes autolesiones y/o ideas suicidas, así como presentes asuntos de la relación y estrés económico. En cambio, las madres estuvieron más propensas a reportar preocupaciones con la crianza tales como el sueño del infante y cómo resolver los desafíos. En general, mucho menos hombres buscaron acceso al apoyo de ParaCuando que las mujeres, a pesar de la conocida prevalencia de preocupaciones de salud mental perinatal entre los papás. También hubo diferencias en cómo los clientes buscaron acceso al servicio, con los papás siendo más a menudo referidos por su pareja íntima, lo que sugiere que las parejas pudieran representar un medio importante para animar a buscar ayuda en el caso de preocupaciones de salud mental perinatal paternas. Estos resultados subrayan la necesidad de adaptar los servicios de salud mental perinatal ‐tradicionalmente diseñados para mujeres‐ para que sean más inclusivos y permitan de mejor manera la participación de hombres.

## 1 INTRODUCTION

Perinatal mental health (PMH) challenges are common among men, with approximately 1 in 10 fathers experiencing poor mental health during pregnancy and the first year after birth (Paulson & Bazemore, 2010; Perinatal Wellbeing Centre, 2019). Men who have a partner facing postnatal depression may be at particular risk, with the rate of paternal postnatal depression as high as 25%–50% among this group (Goodman, 2004). Other risk factors have been identified including previous mental illness, low family income or unemployment, and relationship strain or dissatisfaction (Ansari et al., 2021). Emerging evidence suggests that paternal PMH is associated with a range of negative outcomes including relationship strain (Dudley et al., 2010), negative parent‐child interactions (Giallo et al., 2014), and children's emotional and behavioral challenges (Fletcher et al., 2011). Given the prevalence and potential impacts of paternal PMH issues, it is vital that interventions to effectively support PMH among fathers are available. In light of these issues, there is growing recognition of the need to better support fathers within child and family services, and the development of guides to enhance father‐inclusive practice (Australian Government, 2009; Tehan & McDonald, 2010).

In order to develop PMH interventions that are appropriate and engaging for fathers, it is important to have a clear understanding of the reasons that men might access a PMH support service, and their avenues for help‐seeking. A study examining fathers’ reasons for accessing an Australian perinatal depression and anxiety helpline found that relationship difficulties, infant bonding, and their partner's mental health challenges were common concerns among fathers (Fletcher et al., 2020). Recent research also indicates that external stressors including work‐life balance, changes in the partner relationship, financial instability, and lack of social support, can impact the transition to parenthood and further exacerbate paternal PMH difficulties (Ansari et al., 2021; Ghaleiha et al., 2022; Watkins et al., 2024) and therefore may be an impetus for seeking support, and an important consideration for clinical interventions. Research in this area, however, has been limited, highlighting a need to better understand how and why fathers access PMH services and the ways in which their concerns might overlap with, or differ from, those of mothers.

Another gap lies in understanding gendered differences in help‐seeking for PMH (Reay et al., 2023; Rominov et al., 2018). There is robust evidence showing that men engage in less mental health help‐seeking behaviors than women in general, which contributes to lower treatment rates (e.g., Harris et al., 2016). In the perinatal period, men are frequently excluded from routine PMH screening and report a lack of relevant antenatal education (Reay et al., 2023; Watkins et al., 2024; Wynter et al., 2024), with an estimated 45% of Australian fathers unaware that men can experience perinatal depression as well as women (Perinatal Wellbeing Centre, 2019). Limited engagement with health services during the early years of parenting may present another barrier (Wynter et al., 2024). For example, while fathers might attend some antenatal visits and are usually present at the birth, these interactions typically prioritize mother and baby (Rowe et al., 2013; Watkins et al., 2024). Consequently, fathers may perceive their role as secondary and be reluctant to express a need for support (Darwin et al., 2017). Taken together, research indicates that gender‐specific factors such as traditional masculine norms, lower mental health literacy, self‐stigma, and exclusion from perinatal healthcare settings can all contribute to lower rates of help‐seeking for paternal PMH (Pedersen et al., 2021; Reay et al., 2023; Rominov et al., 2018; Wynter et al., 2024).

**Key findings**

1. Fathers were more likely than mothers to report current self‐harm (SH) or suicidal ideation (SI), with important clinical implications for supporting paternal PMH.
2. Fathers were more likely to report current relationship issues and financial stress, while mothers were more likely to report infant/parenting concerns.
3. Men were more likely than women to be referred by their intimate partner, suggesting that partners may be an important avenue to encourage help‐seeking among fathers facing PMH challenges.

**Statement of relevance**

This research addresses the psychosocial characteristics and presenting concerns of fathers who accessed a service for PMH support. By exploring the unique characteristics of fathers seeking support, this research makes an important contribution to the emerging literature on paternal PMH and provides recommendations to ensure PMH services can connect with fathers and address their needs, supporting them to engage in positive caregiving and promote their infant's healthy development.

Key knowledge gaps remain regarding the distinctive presentations and challenges faced by fathers with PMH concerns, and their help‐seeking behaviors (Wynter et al., 2024). The current study sought to address these gaps by exploring the concerns and presentation of fathers seeking mental health support during the perinatal period, to understand how these might overlap with or differ from maternal PMH concerns. Findings from this research aim to inform the development of inclusive interventions that address the specific needs of fathers, ultimately supporting better family mental health outcomes during this critical period for parents and infants.

## 2 METHODS

### 2.1 Study design

The current study was a retrospective observational analysis of routinely collected ForWhen client data, from the time the service launched on February 1, 2022 to June 10, 2024. In Australia, ForWhen is a national perinatal and infant mental health care navigation service that supports new and expectant parents to access mental health support. ForWhen operates a national phoneline between 9 a.m. and 4:30 p.m., Monday to Friday, staffed by clinical care navigators. Navigators are based in each state and territory of Australia and help connect clients to appropriate support services in their local area (Harris et al., 2024). Navigators conduct mental health screening and psychosocial assessment with clients to identify their level of risk, presenting concerns, and client needs and goals. Navigators record client data on a custom‐built customer relationship management (CRM) system. During the period February 1, 2022 to June 10, 2024, 5008 clients were supported by the ForWhen service, 150 (3%) of whom identified as male. In this paper, we use the terms “men” and “fathers” interchangeably when referring to male clients who accessed the ForWhen service. We use the term “fathers” to include biological fathers, stepfathers, and other father figures. We also acknowledge that not all men in the perinatal period identify as fathers and that some parents who identify as men may have diverse gender experiences.

### 2.2 Measures

The measures reported in this study represent data routinely collected by ForWhen Navigators during intake and assessment procedures with new clients. ForWhen Navigators are skilled clinicians from a range of backgrounds including nursing, social work, and psychology, with expertise and training in perinatal and infant mental health. Navigators use motivational interviewing techniques and a conversational approach to explore clients’ reasons for seeking support, their current needs, risk and protective factors, available support, and goals (see Harris et al., 2024, for more information).

#### 2.2.1 Demographic characteristics

Client demographic information is routinely collected during intake procedures and recorded in the CRM, including: gender, age, location (suburb, postcode, state/territory), Indigenous status, country of birth, antenatal or postnatal status, number of weeks gestation (if pregnant), number of children, age of infant/s.

#### 2.2.2 Kessler psychological distress scale (K10)

The K10 is a validated 10‐item self‐report measure of psychological distress (Kessler et al., 2003). Scores < 19 indicate low distress, 20–24 mild distress, 25–29 moderate distress, and 30–50 indicate severe distress. Navigators administer the K10 to clients as part of routine intake and assessment procedures, to assess current distress symptoms, and record total scores in the CRM.

#### 2.2.3 Mental health and psychosocial characteristics

ForWhen Navigators also collect information on clients’ self‐reported mental health history, presenting concerns, and obstetric and social context risk factors. This information is collected during the initial clinical assessment and entered into the CRM by the ForWhen Navigator (during the call, or immediately afterwards). Data is entered using fixed‐response format items accompanied by free‐text summary boxes (e.g., “history of mental health concerns?”, fixed response answer: “yes/no”; “If yes, provide details”). Mental health history includes any previous or current issues and diagnoses. Clients are always assessed for self‐harm (SH) or suicidal ideation (SI) risk, with safety planning conducted if SH or SI is disclosed. Navigators also explore clients’ obstetric history and childbirth experiences, as well as parenting‐related challenges such as infant settling and sleep difficulties, feeding issues, and bonding or attachment concerns. Social context risk factors are also documented, such as relationship difficulties (e.g., partner conflict, relationship breakdown), social or geographic isolation, financial stress, and other significant life stressors such as bereavement or illness. This information is used to inform referral pathways and the provision of tailored support to meet each client's unique needs.

#### 2.2.4 Family and domestic violence (FDV)

Navigators assess whether current FDV concerns are present. ForWhen adopts a broad definition of FDV that includes any behavior that is violent, threatening, controlling, or intended to make someone feel scared or unsafe. This can include controlling behaviors, physical violence, sexual violence and assault, emotional or psychological abuse, stalking, technology facilitated abuse, and financial abuse. ForWhen Navigators are based in different jurisdictions and use standardized FDV screening tools, such as the New South Wales Health Domestic Violence Routine Screening Tool and Victorian Family Violence Multi‐Agency Risk Assessment and Management Framework, following the requirements of their state or territory legislation. Navigators also ask clients about any experiences of, or exposure to, historic FDV during childhood and/or prior relationships. FDV is assessed both to monitor for any safety concerns for parent and/or infant, and for the purpose of connecting the client with services most appropriate to their current needs and situation. As with data relating to mental health and psychosocial characteristics, ForWhen Navigators record the presence of FDV into the CRM using fixed‐response format items accompanied by details in free‐text summary boxes.

#### 2.2.5 Referral information

For each client, the initial source of their referral (self, family member, health professional) is recorded in the CRM as a fixed‐response item. This is recorded as “self” where the client has made initial contact with the service directly, even if they were recommended to do so by a health professional. When marked as “family member”, this indicates that a family member (usually a partner) made initial contact with ForWhen, and the Navigator subsequently made direct contact with the client. If marked as “health professional”, this means ForWhen received a referral from another clinician or service (e.g., midwife, general practitioner) and subsequently made direct contact with the client. In some cases, clients contact the service to seek support not only for themselves, but also for their partner. Where this is the case, it is recorded by Navigators in the CRM, either as part of the assessment summary, through the creation of a linked case (i.e., where both partners are clients of ForWhen), and/or through recorded Navigator actions (e.g., father‐specific resources provided to a female client, to give to their partner).

### 2.3 Procedure

Data were extracted from the ForWhen CRM for two groups: (i) male clients, and (ii) a comparison group of female clients. The male sample comprised all male clients who accessed the ForWhen service during the study period, were seeking PMH support for themselves, and who had engaged in an intake triage and assessment phone call. Data records were excluded for male callers/referrals who: had accessed the ForWhen service seeking support for their partner only; did not engage with the service after their initial referral; and/or were out of scope (e.g., no current mental health concerns) (Table 1). These individuals were excluded as they did not complete intake assessments with Navigators in relation to themselves, and therefore adequate data were not available.

The comparison group included 203 female clients who participated in a separate study examining clinical outcomes of the ForWhen service (Kohlhoff et al., under, review). In that study, 212 ForWhen clients consented to baseline and follow‐up questionnaires, and their CRM data records were extracted. Of these 212 participants, 203 were female and formed the comparison group for the present study. These clients had accessed the ForWhen service between January 1, 2023 and August 8, 2023; were seeking PMH support for themselves; and had engaged in an intake triage and screening phone call.

Data relating to client demographic characteristics, level of distress (K10 score; Kessler et al., 2003), mental health history, current or historic FDV, obstetric risk factors (e.g., birth trauma, previous loss), social context risk factors (e.g., social/geographic isolation, current relationship issues), parenting concerns (e.g., sleep and settling, parent‐infant bonding), and ForWhen referral information were extracted from the ForWhen CRM database. ForWhen Navigators gathered this information from each client as a routine part of their initial assessment and entered it into the CRM using a series of fixed‐responses and free‐text summaries. A researcher reviewed the fixed‐responses and free‐text summaries and allocated codes to each client, to identify whether or not each characteristic was present. All extracted data was self‐reported by clients and recorded in the CRM by ForWhen Navigators—as such, the data represent the concerns reported by clients and noted by Navigators during intake, and are not necessarily exhaustive.

### 2.4 Participants

#### 2.4.1 Male sample

**FIGURE 1:**
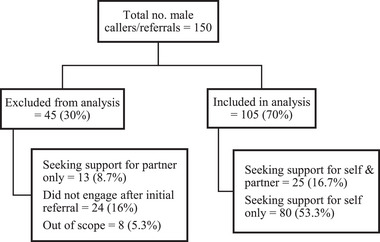
Male client data retained for analysis.

Of the 150 male callers to the ForWhen service during the given time‐period (February 1, 2022 to June 10, 2024), 45 were excluded and 105 were retained for analysis (see Figure 1). Demographic characteristics are shown in Table 1. Of the 105 males who were included in the analysis, the mean age was 33.8 years (range 17.9–53.1, SD = 6.46). Male participants lived across all states and territories of Australia; 28.6% in Victoria, 21.9% in Western Australia, 12.4% in Queensland, 11.4% in Tasmania, 9.5% in New South Wales, 8.6% in South Australia, 4.8% in Australian Capital Territory, and 2.9% in the Northern Territory. At the time of program entry, 18.1% were currently pregnant/expecting, and 81.9% were in the postnatal period (up to 12 months after birth). A total of 68.6% were first‐time parents. According to modified monash model (MM) classifications of client postcodes, 75.8% lived in metropolitan areas (MM 1), 16.2% lived in regional areas (MM 2–3), and 8.1% lived in rural or remote areas (MM 4–7) (Australian Government Department of Health, 2019). According to the Index of Relative Socio‐economic Advantage and Disadvantage (IRSAD), 8.1% were classified in the lowest 20th percentile for socio‐economic disadvantage (most disadvantaged) and 28.3% were in the top 20th percentile (most advantaged) (Australian Bureau of Statistics, 2021).

**TABLE 1:** Demographic information for male and female client samples.

Demographic variable	Male clients *N* = 105	Female clients *N* = 203	Between group comparison
	*n* (%)	*n* (%)	*p value*
Rurality^*^			.657
Metropolitan area	75 (75.8)	163 (80.3)	
Regional area	16 (16.2)	26 (12.8)	
Rural or remote area	8 (8.1)	14 (6.9)	
Socio‐economic disadvantage (percentile)*			.290
0–20	8 (8.1)	26 (12.8)	
21–40	20 (20.2)	24 (11.8)	
41–60	19 (19.2)	42 (20.7)	
61–80	24 (24.2)	46 (22.7)	
81–100	28 (28.3)	65 (32.0)	
Aboriginal or Torres Strait Islander	2 (1.9)	7 (3.4)	.446
Born overseas	19 (18.1)	47 (23.2)	.305
First‐time parent (pre or postnatal)	72 (68.6)	139 (68.5)	.986
Age of infant			.698
Antenatal	19 (18.1)	29 (14.3)	
0–3 months	49 (46.7)	84 (41.4)	
3–6 months	20 (19.0)	43 (21.2)	
6–9 months	8 (7.6)	22 (10.8)	
9–12 months	7 (6.7)	21 (10.3)	
12+ months	2 (1.9)	4 (2.0)	

* Based on client postcode data available for 99/105 male clients and 203/203 female clients.

#### 2.4.2 Female sample

Of the 203 female clients who comprised the comparison group, the mean age was 32.59 years (SD = 4.72, Range 17.6–43.8). Female participants lived across all states and territories of Australia; 20.7% in Victoria, 19.2% in Queensland, 18.7% in South Australia, 15.8% in Western Australia, 12.3% in New South Wales, 4.9% in Northern Territory, 4.4% in Australian Capital Territory, and 3.9% in Tasmania. At the time of program entry, 14.3% were currently pregnant/expecting, and 85.7% were in the postnatal period (up to 12 months after birth). A total of 68.5% were first‐time parents. According to MM classifications of client postcodes, 80.3% lived in metropolitan areas (MM 1), 12.8% lived in regional areas (MM 2–3), and 6.9% lived in rural or remote areas (MM 4–7) (Australian Government Department of Health, 2019). According to the IRSAD, 12.8% were classified in the lowest 20th percentile for socio‐economic disadvantage (most disadvantaged) and 32% were in the top 20th percentile (most advantaged) (Australian Bureau of Statistics, 2021).

### 2.5 Ethical approval

This research was approved by South Western Sydney Local Health District Human Research Ethics Committee (2021/ETH11611).

### 2.6 Data analysis

Data were analyzed using basic descriptive statistics. Chi‐square tests of independence were conducted to examine the association between gender (male vs. female), and demographic characteristics, mental health history, psychosocial risk factors and parenting concerns, and referral information relating to the client groups. Statistical significance was set at .05; all analyses were conducted in SPSS version 28.

### 2.7 Findings

#### 2.7.1 Demographic characteristics

There were no significant differences on any demographic variables between male and female client groups (*p*s > .05; Table 1). Both groups included similarly high proportions of first‐time parents (pre or postnatal); 68.6% of male clients and 68.5% of the female comparison group, respectively (*p *> .05). There were no significant differences between groups in terms of being antenatal/postnatal or infant age (*p*s > .05; Table 1); with 18.1% of male clients and 14.3% of female clients presenting in the antenatal period. For both groups, most clients had babies between 0 and 6 months of age; 65.7% for male clients and 62.6% for female clients.

#### 2.7.2 Current distress and mental health history

Male and female clients did not significantly differ in level of distress on admission to ForWhen (as measured by the K10, administered by Navigators, *p *> .05). Male clients were, however, significantly more likely to report current SH or SI χ^2^ (1308) = 4.351, *p* = .037, than were female clients (Table 2). Male and female clients did not significantly differ in the rate of reported mental health history (*p *> .05), with 72.4% of men and 67.2% of women reporting any previous mental health diagnoses or challenges. However, female clients were significantly more likely to report a history of anxiety disorders, *χ* ^2^ (1, 308) = 4.433, *p* = .035, while male clients were significantly more likely to report a history of substance abuse, χ^2^ (1, 308) = 21.583, *p *= < .001.

**TABLE 2:** Mental health and psychosocial characteristics of male and female client samples.

Variable	Male clients *N* = 105	Female clients *N* = 203	Between group comparison
	*n* (%)	*n* (%)	*p value*
K10 distress score on entry^a^			.543
10–24 (mild)	16 (22.9)	53 (29.3)	
25–29 (moderate)	19 (27.1)	41 (22.7)	
30–50 (severe)	35 (50.0)	87 (48.0)	
Current self‐harm or suicidal ideation (SH/SI)	30 (28.6)	37 (18.2)	**.037** ^*^
Mental health history			
History of depression	51 (48.6)	78 (38.4)	.087
History of anxiety	36 (34.2)	95 (46.8)	**.035** ^*^
History of substance abuse	18 (17.1)	5 (2.5)	**<.001** ^*^
*Any significant mental health history*	76 (72.4)	136 (67.2)	.333
Family and domestic violence (FDV)			
Historic FDV	19 (18.1)	32 (15.8)	.602
Current FDV	5 (4.8)	16 (7.9)	.303
*Any FDV* ^b^	19 (18.1)	39 (19.2)	.812
Social context risk factors			
Social or geographical isolation	38 (36.1)	74 (36.5)	.964
Relationship issues	42 (40.0)	51 (25.1)	.007*
Financial stress	20 (19.0)	21 (10.3)	.033*
*At least one social context risk factor*	65 (61.9)	107 (52.7)	.123
Obstetric risk factors			
Birth trauma	23 (21.9)	46 (22.7)	.880
Previous loss or termination	6 (5.7)	18 (8.9)	.328
Fertility issues	3 (2.9)	14 (6.9)	.141
Twins	6 (5.7)	10 (4.9)	.768
Unplanned pregnancy	5 (4.8)	13 (6.4)	.560
*At least one obstetric risk factor*	36 (34.2)	79 (38.9)	.426
Parenting concerns			
Infant sleep or settling challenges	14 (13.3)	52 (25.6)	**.013** ^*^
Infant feeding challenges	5 (4.8)	20 (9.9)	.121
Parent‐infant bonding concerns	13 (12.4)	23 (11.3)	.786
*At least one parenting concern*	28 (26.7)	78 (38.4)	**.040** ^*^

Abbreviation: FDV, family and domestic violence.

^a^ Admission K10 data available for 70/105 male clients and 181/203 female clients.

^b^ Number of clients who reported exposure to either current or historic FDV, or both.

* indicates a statistically significant result.

#### 2.7.3 FDV and social context risk factors

Male and female clients did not differ in the rate of reported historic or current experiences of FDV (*ps *> .05), with men reporting rates of any FDV exposure at 18.1% and women at 19.2% (Table 2). Similarly, social and/or geographic isolation was high across both groups (*p *> .05), reported by 36.1% of men and 36.5% of women. Male clients were however significantly more likely to report relationship issues, *χ* ^2^ (1, 308) = 7.267, *p* = .007, with 40% of men and 25.1% of women reporting current challenges in their intimate partner relationship. Men were also significantly more likely than women to report financial stress or worries, *χ* ^2^ (1, 308) = 4.542, *p* = .033, with 19% of men reporting financial concerns compared to 10.3% of women.

#### 2.7.4 Obstetric risk factors and parenting concerns

There were no significant differences between male and female clients in the rate they reported any of the identified obstetric risk factors, including birth trauma, previous pregnancy loss or termination, fertility issues, twin births, or unplanned pregnancy (*ps *> .05; Table 2). For both groups birth trauma was relatively common, reported by 21.9% of male clients and 22.7% of female clients, respectively. Male and female clients significantly differed in how likely they were to report any parenting concern (e.g., infant sleep or feeding), *χ* ^2^ (1, 308) = 4.224, *p* = .040, with 38.4% of women reporting at least one parenting concern compared to 26.7% of men. In particular, women were significantly more likely than men to report infant sleep and/or settling challenges, *χ* ^2^ (1, 308) = 6.201, *p* = .013. However, both groups reported similar rates of parent‐to‐infant bonding concerns (*p *> .05); 12.4% of men and 11.3% of women, respectively.

#### 2.7.5 Referral information

Client records were examined to compare the source of initial referral between male and female client samples—that is, whether the initial contact with the ForWhen service came from the client themselves (self‐referral), an intimate partner (partner‐initiated referral), or a health professional (clinician‐initiated referral; Table 3). Results showed significant differences between groups, *χ* ^2^ (2, 308) = 42.848, *p* = > .001, with male clients more likely to be referred by a health professional (31.4% vs. 24.6%), and by an intimate partner (19% vs. 0.5%). Conversely, female clients were much more likely to self‐refer to the service (74 .9% vs. 49.5%). Male and female clients also differed in whether they were seeking support for themselves only, or whether they also sought support for their partner *χ* ^2^ (1, 308) = 26.457, *p* = > .001. Among the men, 23.8% were also seeking support for their partner, compared to only 4.4% of the women (Table 3).

**TABLE 3:** Referral information for male and female client samples.

Referral information	Male clients *N* = 105	Female clients *N* = 203	Between group comparison
	*n* (%)	*n* (%)	*p value*
Who initiated the referral			**>.001** ^*^
Client (self‐referral)	52 (49.5)	152 (74.9)	
Intimate partner	20 (19.0)	1 (0.5)	
Health professional	33 (31.4)	50 (24.6)	
Seeking support for			**>.001** ^*^
Self only	80 (76.2%)	194 (95.6%)	
Self and partner	25 (23.8%)	9 (4.4%)	

* indicates a statistically significant result.

## 3 DISCUSSION

This study examined the demographic and clinical presentations of male and female clients of a PMH care navigation service, with the aim of examining gender‐based differences influencing mental health concerns, risk factors, and help‐seeking behaviors. The findings shed light on common and unique needs of fathers and mothers and the relational nature of PMH.

Both male and female clients shared similar demographic profiles. The high proportion of clients with significant mental health history across both groups supports existing literature showing that a history of mental illness is a major risk factor for developing PMH issues. The majority of both fathers and mothers were first‐time parents, suggesting that those becoming parents for the first time may also be at heightened risk for PMH challenges, regardless of gender. However, mental health history revealed gendered differences—men were more likely to report a history of substance abuse while women more frequently reported anxiety—broadly aligning with prior research on gender differences in mental health which likely carry over to the perinatal period (Farhane‐Medina et al., 2022).

While fathers and mothers did not differ in the severity of their distress on intake, men were much more likely to report current SH and SI than women. The prevalence of SH/SI among our sample of mothers corresponds with previous research on SH/SI among postpartum women with mood disorders (Pope et al., 2013) and constitutes a significant risk. Concerningly, we found even higher rates among our sample of fathers—with almost 1 in 3 disclosing SH/SI. A previous study by Quevedo et al. (2011) investigating suicide risk among postnatal fathers also reported high rates, particularly among fathers who had depression, anxiety, or mixed episodes of mania/depression. These findings highlight an urgent need to examine paternal suicide risk within the perinatal period, an area which has not been adequately explored, and the need for adequate screening and interventions to support vulnerable fathers.

For some fathers, witnessing a traumatic birth can bring about long‐lasting distress and negative thoughts (Hughes et al., 2020; White, 2007). Very little research exists regarding the prevalence of birth trauma among fathers and nonbirth partners, yet almost 1 in 5 fathers in our study reported birth trauma during their intake assessment. Interestingly, the rates of reported birth trauma were similar among men and women, underlining the relevance of birth trauma in assessments of paternal, as well as maternal, PMH. However, a limitation was that birth trauma was recorded during intake assessment if the client raised it as a significant concern, or if their child's birth was described as “traumatic” when they were asked about it. This does not necessarily entail symptoms of ongoing trauma or distress directly related to the birth experience. On the other hand, birth trauma may have been underreported, as the figures are based on client self‐report during initial psychosocial assessment and are not necessarily exhaustive. Further research using validated measures is needed to understand the prevalence of birth trauma and its impact on fathers’, as well as mothers’, postnatal wellbeing.

Our findings regarding financial concerns among fathers provide insight into unique stressors that men may encounter during the perinatal period. As highlighted in prior research on new fatherhood, men often retain primary responsibility for employment and income during the postpartum period, and may face heightened financial pressures that compound PMH risks (Cooklin et al., 2015). Fathers are often balancing the demands of employment with new responsibilities at home, and may face pressure in maintaining work‐life balance and caregiving responsibilities, contributing to relationship strain (Cooklin et al., 2015). Conversely, women in our sample more frequently reported concerns around parenting concerns, such as infant sleep and settling challenges, reflecting traditional gendered divisions in caregiving labor where women are more likely to be the infant's primary caregiver. Understanding how contextual gendered differences shape the unique expectations and stressors faced by mothers and fathers is essential for tailoring support services to the needs of each parent and for promoting equitable parenting responsibilities.

A significant proportion (23.8% of included) of male clients were seeking support not only for themselves, but also for their female partner. Interestingly this was not true for the female client group, suggesting that men's mental health may be particularly vulnerable where their partner is struggling. Also, men frequently reported strain in their intimate partner relationship (40%), as did about 25% of women. While it is not possible to know if/how these factors directly contributed to wellbeing, findings do point to the dyadic nature of PMH, in that partners’ mental health is often interconnected, and that relationship quality may be vulnerable where one or both partners experience poor PMH (Goodman, 2004). Reported relationship strain was significantly higher among men than women, supporting previous research linking relationship difficulties/breakdown to increased PMH in fathers in particular (Ansari et al., 2021; Dudley et al., 2010).

These findings have a range of clinical implications, including the need for couple/relationship‐focused PMH support and for clinicians to enquire about relationship wellbeing when assessing clients during the perinatal period. Given the prevalence of relationship strain in those at risk of PMH challenges, comprehensive antenatal education should include psychoeducation about the impact of new parenthood on intimate relationships, and provide strategies for navigating relationship challenges during the perinatal period such as tools for effective communication and conflict resolution. Routine screening for PMH disorders could also be extended to fathers, particularly in cases where their partner has been identified as experiencing poor PMH. Finally, clinical interventions for PMH problems should aim to support effective communication and strategies to improve relationship quality and satisfaction, incorporating a relational lens (Rominov et al., 2018).

Finally, our findings indicate significant gendered difference in help‐seeking behaviors for PMH. Compared to roughly 3/4 of female clients, less than half of male clients made the initial contact with ForWhen themselves. Fathers were much more likely to access the PMH care navigation service after being referred by someone else—such as a health professional, but especially by an intimate partner—highlighting how a female partner may be an important avenue to encourage help‐seeking behaviors among men (Rominov et al., 2018) and a main source of emotional support during the perinatal period (Ghaleiha et al., 2022). There were also a significant number of male clients who did not follow through with intake assessment after having been referred to ForWhen (16%), indicating possible hesitancy to engage with support even after PMH issues have been identified. However, we did not have a comparative figure for the sample of female clients, so it is difficult to know if men are less likely to engage in support than women. The finding that men were less likely to initiate their own referral (i.e., call into the phoneline directly) broadly supports other research reporting lower rates of help‐seeking for PMH challenges among fathers (Wynter et al., 2024), a phenomenon that has been attributed to barriers including masculine stereotypes, self‐stigma, and lack of awareness regarding paternal PMH (Addis & Mahalik, 2003; Mansfield et al., 2005; Rominov et al., 2018; Wynter et al., 2024).

The overall proportion of male ForWhen clients stands in contrast to the known prevalence of paternal PMH concerns (Paulson & Bazemore, 2010; Perinatal Wellbeing Centre, 2019). Another study of fathers supported by an Australian PMH service similarly reported a small proportion of male clients (Fletcher et al., 2020), highlighting the need for PMH services to be more effective in engaging fathers. This discrepancy suggests that existing PMH service models may not be designed or promoted in ways that resonate with fathers. Addressing this requires both service‐level changes to improve father‐inclusivity, and a broader cultural shift toward recognizing paternal PMH challenges and fostering openness to seeking and providing support.

## 4 LIMITATIONS AND FUTURE DIRECTIONS

A key limitation of this study is that the data come from psychosocial assessments and case notes recorded by ForWhen Navigators during intake procedures. Therefore, they may capture only partial insights about client mental health history, risk factors, or current concerns, depending on what clients chose to disclose during those conversations. However, a strength was that the intake assessments were undertaken by a clinical Navigator with professional expertise in PMH, trained in conducting mental health and risk assessment and engagement interviewing techniques to understand the nature and scope of clients’ concerns and record these appropriately. Further research on the prevalence and nature of PMH concerns among fathers could use standardized measures to overcome this limitation.

Another limitation was the difference in recruitment methods between the male sample and the female comparison group. The female sample comprised clients who had consented to participate in a separate study—this approach was chosen because data for these clients had already been collected and coded, enabling efficient and consistent comparison. However, it presents the potential for sampling bias as clients who agreed to research participation and follow‐up may differ from those who did not. Further, the overall sample of men who engaged with the service was small (3% of all clients) given the known prevalence of PMH challenges among men, and may not be representative of the broader population of fathers in need of support. For instance, their willingness to engage with the service could be due to higher levels of distress or greater openness to help‐seeking compared to other fathers. Further research is needed to explore access barriers for men and how PMH services can be adapted to better engage fathers. A final limitation related to the data around FDV. Unfortunately, we did not have access to sufficient data to reliably report on whether each client was an aggressor, victim, or both. Future work is required to better explore and understand these nuances.

This study provides valuable insights in the distinct, and overlapping, needs and concerns of both fathers and mothers seeking support from a PMH care navigation service. The high prevalence of SH and SI among fathers, gender‐specific help‐seeking behaviors, as well as the observed associations between gender and varied social context stressors, underscore the importance of research that investigates paternal PMH specifically, as well as informing father‐inclusive PMH services that effectively engage and support new and expectant fathers. Addressing these areas through further research and clinical practice will be critical to providing comprehensive PMH care to effectively support both men and women in their parenting role and wellbeing, which will ultimately also be of significant benefit to their children.

## CONFLICT OF INTEREST STATEMENT

The authors have no competing interests to report.

## References

[imhj70012-bib-0001] Addis, M. E. , & Mahalik, J. R. (2003). Men, masculinity, and the contexts of help seeking. American Psychologist, 58(1), 5. 12674814 10.1037/0003-066x.58.1.5

[imhj70012-bib-0002] Ansari, N. S. , Shah, J. , Dennis, C. L. , & Shah, P. S. (2021). Risk factors for postpartum depressive symptoms among fathers: A systematic review and meta‐analysis. Acta Obstetricia et Gynecologica Scandinavica, 100(7), 1186–1199. 33539548 10.1111/aogs.14109

[imhj70012-bib-0003] Australian Bureau of Statistics . (2021). Socio‐Economic Indexes for Areas (SEIFA) .

[imhj70012-bib-0004] Australian Government . (2009). Father‐Inclusive Practice Guide . Retrieved from https://www.opgroeien.be/sites/default/files/tool‐documents/father_inclusive_practice.pdf

[imhj70012-bib-0005] Australian Government Department of Health . (2019). Modified Monash Model Fact Sheet .

[imhj70012-bib-0006] Cooklin, A. R. , Giallo, R. , Strazdins, L. , Martin, A. , Leach, L. S. , & Nicholson, J. M. (2015). What matters for working fathers? Job characteristics, work‐family conflict and enrichment, and fathers' postpartum mental health in an Australian cohort. Social Science & Medicine, 146, 214–222. 26520473 10.1016/j.socscimed.2015.09.028

[imhj70012-bib-0007] Darwin, Z. , Galdas, P. , Hinchliff, S. , Littlewood, E. , McMillan, D. , McGowan, L. , & Gilbody, S. (2017). Fathers' views and experiences of their own mental health during pregnancy and the first postnatal year: A qualitative interview study of men participating in the UK Born and Bred in Yorkshire (BaBY) cohort. BMC Pregnancy Childbirth, 17(1), 45. 10.1186/s12884-017-1229-4 28125983 PMC5270346

[imhj70012-bib-0008] Dudley, M. , Roy, K. , Kelk, N. , & Bernard, D. (2010). Psychological correlates of depression in fathers and mothers in the first postnatal year. Journal of Reproductive and Infant Psychology, 19(3), 187–202.

[imhj70012-bib-0009] Farhane‐Medina, N. Z. , Luque, B. , Tabernero, C. , & Castillo‐Mayén, R. (2022). Factors associated with gender and sex differences in anxiety prevalence and comorbidity: A systematic review. Science Progress, 105(4), 00368504221135469. 10.1177/00368504221135469 36373774 PMC10450496

[imhj70012-bib-0010] Fletcher, R. , StGeorge, J. , Newman, L. , & Wroe, J. (2020). Male callers to an Australian perinatal depression and anxiety help line—Understanding issues and concerns. Infant Mental Health Journal, 41(1), 145–157. 31524292 10.1002/imhj.21829

[imhj70012-bib-0011] Fletcher, R. J. , Feeman, E. , Garfield, C. , & Vimpani, G. (2011). The effects of early paternal depression on children's development. Medical Journal of Australia, 195(11–12), 685–689. 22171866 10.5694/mja11.10192

[imhj70012-bib-0012] Ghaleiha, A. , Barber, C. , Tamatea, A. J. , & Bird, A. (2022). Fathers’ help seeking behavior and attitudes during their transition to parenthood. Infant Mental Health Journal, 43(5), 756–768. 35913697 10.1002/imhj.22008 PMC9542128

[imhj70012-bib-0013] Giallo, R. , Cooklin, A. , Wade, C. , D'Esposito, F. , & Nicholson, J. M. (2014). Fathers’ postnatal mental health and child well‐being at age five: The mediating role of parenting behavior. Journal of Family Issues, 35(11), 1543–1562. 10.1111/cch.12028 23363326

[imhj70012-bib-0014] Goodman, J. H. (2004). Paternal postpartum depression, its relationship to maternal postpartum depression, and implications for family health. Journal of Advanced Nursing, 45(1), 26–35. 14675298 10.1046/j.1365-2648.2003.02857.x

[imhj70012-bib-0015] Harris, M. , Baxter, A. , Reavley, N. , Diminic, S. , Pirkis, J. , & Whiteford, H. (2016). Gender‐related patterns and determinants of recent help‐seeking for past‐year affective, anxiety and substance use disorders: Findings from a national epidemiological survey. Epidemiology and Psychiatric Sciences, 25(6), 548–561. 26428069 10.1017/S2045796015000876 PMC7137666

[imhj70012-bib-0016] Harris, S. A. , Eapen, V. , & Kohlhoff, J. (2024). Implementing a national navigation service for perinatal and infant mental health: Early learnings from the ForWhen model. Community Mental Health Journal, 60, 581–588. 10.1007/s10597-023-01211-0 37991577

[imhj70012-bib-0017] Hughes, C. , Foley, S. , Devine, R. T. , Ribner, A. , Kyriakou, L. , Boddington, L. , Holmes, E. A. , & NewFAMS Team Creators/Copyright Holders & NewFAMS Team & Contributors . (2020). Worrying in the wings? Negative emotional birth memories in mothers and fathers show similar associations with perinatal mood disturbance and delivery mode. Archives of Women's Mental Health, 23(3), 371–377. 10.1007/s00737-019-00973-5 PMC7244466 31280385

[imhj70012-bib-0018] Kessler, R. C. , Barker, P. R. , Colpe, L. J. , Epstein, J. F. , Gfroerer, J. C. , Hiripi, E. , Howes, M. J. , Normand, S.‐L. T. , Manderscheid, R. W. , Walters, E. E. , & Zaslavsky, A. M. (2003). Screening for serious mental illness in the general population. Archives of General Psychiatry, 60(2), 184–189. 12578436 10.1001/archpsyc.60.2.184

[imhj70012-bib-0019] Kohlhoff, J. , Harris, S. A. , Traynor, N. , Vorgias, J. , & Eapen, V. (unpublished). Clinical outcomes of a perinatal and infant mental health care navigation service [manuscript in preparation].

[imhj70012-bib-0020] Mansfield, A. K. , Addis, M. E. , & Courtenay, W. (2005). Measurement of men's help seeking: Development and evaluation of the barriers to help seeking scale. Psychology of Men & Masculinity, 6(2), 95–108.

[imhj70012-bib-0021] Paulson, J. F. , & Bazemore, S. D. (2010). Prenatal and postpartum depression in fathers and its association with maternal depression: A meta‐analysis. Journal of the American Medical Association, 303(19), 1961–1969. 20483973 10.1001/jama.2010.605

[imhj70012-bib-0022] Pedersen, S. C. , Maindal, H. T. , & Ryom, K. (2021). “I Wanted to be there as a father, but i couldn't”: A qualitative study of fathers' experiences of postpartum depression and their help‐seeking behavior. American Journal of Men's Health, 15(3), 15579883211024375. 10.1177/15579883211024375 PMC8202277 34116610

[imhj70012-bib-0023] Perinatal Wellbeing Centre . (2019). The cost of perinatal depression and anxiety in Australia . https://www.perinatalwellbeingcentre.org.au/Handlers/Download.ashx?IDMF=53aab8d3‐c748‐4818‐abab‐32a58d3c510f

[imhj70012-bib-0024] Pope, C. J. , Xie, B. , Sharma, V. , & Campbell, M. K. (2013). A prospective study of thoughts of self‐harm and suicidal ideation during the postpartum period in women with mood disorders. Archives of Women's Mental Health, 16(6), 483–488. 10.1007/s00737-013-0370-y 23784481

[imhj70012-bib-0025] Quevedo, L. , da Silva, R. A. , Coelho, F. , Pinheiro, K. A. T. , Horta, B. L. , Kapczinski, F. , & Pinheiro, R. T. (2011). Risk of suicide and mixed episode in men in the postpartum period. Journal of Affective Disorders, 132(1), 243–246. 10.1016/j.jad.2011.01.004 21277023

[imhj70012-bib-0026] Reay, M. , Mayers, A. , Knowles‐Bevis, R. , & Knight, M. T. (2023). Understanding the barriers fathers face to seeking help for paternal perinatal depression: Comparing fathers to men outside the perinatal period. International Journal of Environmental Research and Public Health, 21(1), 16. 38276804 10.3390/ijerph21010016 PMC10815257

[imhj70012-bib-0027] Rominov, H. , Giallo, R. , Pilkington, P. D. , & Whelan, T. A. (2018). “Getting help for yourself is a way of helping your baby:” Fathers' experiences of support for mental health and parenting in the perinatal period. Psychology of Men & Masculinity, 19(3), 457.

[imhj70012-bib-0028] Rowe, H. J. , Holton, S. , & Fisher, J. R. (2013). Postpartum emotional support: A qualitative study of women's and men's anticipated needs and preferred sources. Australian Journal of Primary Health, 19(1), 46–52. 22951012 10.1071/PY11117

[imhj70012-bib-0029] Tehan, B. , & McDonald, M. (2010). Engaging fathers in child and family services. Australian Institute of Family Studies. https://aifs.gov.au/sites/default/files/publication‐documents/ps2_0.pdf

[imhj70012-bib-0030] Watkins, A. E. , El Zerbi, C. , McGovern, R. , & Rankin, J. (2024). Exploration of fathers’ mental health and well‐being concerns during the transition to fatherhood, and paternal perinatal support: Scoping review. BMJ Open, 14(11), e078386. 10.1136/bmjopen-2023-078386 PMC11574476 39532383

[imhj70012-bib-0031] White, G. (2007). You cope by breaking down in private: Fathers and PTSD following childbirth. British Journal of Midwifery, 15(1), 39–45.

[imhj70012-bib-0032] Wynter, K. , Mansour, K. A. , Forbes, F. , & Macdonald, J. A. (2024). Barriers and opportunities for health service access among fathers: A review of empirical evidence. Health Promotion Journal of Australia, 35(4), 891–910. 10.1002/hpja.846 38494641

